# Modelling reoxygenation effects in non-small cell lung cancer cell lines and showing epithelial-mesenchymal transition

**DOI:** 10.1007/s00432-022-04242-4

**Published:** 2022-08-06

**Authors:** Joanna Kapeleris, Juliana Müller Bark, Shanon Ranjit, Derek Richard, Ian Vela, Kenneth O’Byrne, Chamindie Punyadeera

**Affiliations:** 1 Centre for Biomedical Technologies, School of Biomedical Sciences, Faculty of Health, Queensland University of Technology, 60 Musk Avenue, GPO Box 2434, Kelvin Grove, QLD 4059 Australia; 2 Translational Research Institute, Woolloongabba, Brisbane, Australia; 3 Cancer & Ageing Research Program, Queensland University of Technology, Translational Research Institute, Woolloongabba, Brisbane, Australia; 4 Australian Prostate Cancer Research Centre, Queensland University of Technology, Princess Alexandra Hospital, Translational Research Institute, Brisbane, QLD Australia; 5 Princess Alexandra Hospital, Woolloongabba, QLD Australia; 6 Saliva and Liquid Biopsy Translational Laboratory, Griffith Institute for Drug Discovery, Griffith University, 46 Don Yong Road, Nathan, Brisbane, Australia; 7 Menzies Health Institute Queensland, Griffith University, Gold Coast, Australia

**Keywords:** Non-small cell lung cancer, Rare cells, Hypoxia, Reoxygenation, Epithelial-mesenchymal transition, Circulating tumour cell

## Abstract

**Purpose:**

Circulating tumour cells (CTCs) are a rare cell subpopulation regulated by the tumour microenvironment. In hypoxic conditions, CTCs are able to invade the lymphatic and circulatory systems leading to metastasis at distant sites.

**Methods:**

To mimic in vivo oxygen variations and effects on CTCs, we have cultured five non-small cell lung cancer (NSCLC) cell lines under normoxic and hypoxic conditions, followed by a pulse of reoxygenation for 4 h.

**Results:**

Proliferation, spheroid-formation and colony formation ability under varying O_2_ levels were investigated. Proliferation rate was not altered when cells were cultured in 2D models under hypoxic conditions. However, we observed that hypoxia enhanced in vitro formation of tumour-spheres and accelerated clonogenicity of NSCLC cell lines. In addition, cells exposed to hypoxia and reoxygenation conditions showed altered expression of epithelial-mesenchymal transition (EMT) related genes in NSCLC cell lines both at mRNA (AKT1, CAMK2NH1, DESI1, VIM, MAP1B, EGFR, ZEB1, HIF1α) and protein levels (Vimentin, Pan-cytokeratin).

**Conclusion:**

Our data suggest that when investigating CTCs as a prognostic biomarker in NSCLC, it is also essential to take into consideration EMT status to obtain a comprehensive overview of CTCs in circulation.

## Introduction

Metastasis is an extremely complex, multistep process (Alizadeh et al. 2014). Cells must gain the ability to intravasate into the bloodstream from the bulk tumour, travel through the circulation, undergo sheer stressors and escape immune evasion, and extravasate to favourable metastatic sites such as bone, brain and liver (Weiss 2003; Maheswaran and Haber 2010; Hess et al. 2006). To detach from a primary tumour and disseminate into blood, tumour cells must undergo a cellular process known as epithelial-mesenchymal transition (EMT) (Thiery 2002; Nieto et al. 2016; Yang et al. 2020).

EMT enables tumour cells to become motile, leading to enhancing migratory capabilities, allowing cells to penetrate into the lymph vasculature and circulate as single or clusters of circulating tumour cells (CTCs) (Nurwidya et al. 2016). Whilst in blood, CTCs exist in a dynamic EMT status (Yu et al. 2013). CTCs extravasate and colonise at distant organs, undergoing mesenchymal to epithelial transition (MET, a reverse process) (Tsai and Yang 2013). EMT is thought to support cell invasiveness but restrict proliferation, thereby maintaining cancer cell survival in metastatic sites, whereas MET re-activates proliferative potential (Ocaña Oscar et al. 2012). The famous “seed and soil” hypothesis proposed by Stephen Pagent in the nineteenth century suggests that tumour cells (the ‘seed’) have a preference to metastasise to certain organs (the ‘soil’) (Paget 1989). This hypothesis has since been revisited by Fidler and Langly, still holding significance in cancer research today (Fidler 2003; Langley and Fidler 2011).

Cancer cells utilise several means to escape immune surveillance in the blood (Messerschmidt et al. 2016). One immune evasion mechanism is where CTCs adapt to hypoxia, thereby allowing cancer cells to “hide” in hypoxic cellular niches where immune effector functions are weakened. This oxygen fluctuation activates survival pathways and is thought to contribute to cancer cell resistance to chemo and radiotherapies (Semenza 2009; Noman et al. 2014; Onnis et al. 2009; Colliez et al. 2017; Hill et al. 2015; Bittner and Grosu 2013). Oxygenation is a vital component of the tumour microenvironment and is a significant contributing factor in cancer progression (Vaupel and Mayer 2007). Expansion of cancer cells during tumour development promotes a hypoxic environment followed by reoxygenation to promote tumour progression. CTCs released from a hypoxic environment will experience a pulse of reoxygenation (Hong et al. 2016).

Tumour hypoxia has been recognised as a contributor to resistance to radiotherapy. Furthermore, it has been thought that tumour oxygenation has been shown to correlate with the outcome of patients undergoing radiation therapies (Colliez et al. 2017). More recently, strategies have also been developed to alleviate tumour hypoxia to radiosensitise tumours (Colliez et al. 2017). The lack of biomarkers, as well as inadequate methods for the assessment of tumour hypoxia, prevents hypoxia from being used as a potential “biomarker” to stratify the risk and to select patients who could benefit through targeted therapies. The discovery of hypoxia-inducible factors (HIFs) that mediate transcriptional responses to altering oxygen levels has shown promise as a targeted treatment (Majmundar et al. 2010). Hypoxic signalling through HIF has shown to be a vital trigger and a modulator of EMT (Thiery and Sleeman 2006). It is known that hypoxia and HIF factors can directly increase the expression of cancer stem cell (CSC) markers and induce CSC properties. As such, one would predict that hypoxia and HIFs might contribute to CTC generation and CTC CSC-like phenotype resistant to the immune system (Noman et al. 2014).

Hypoxia enhances cancer cell survival and metastasis in numerous tumour types; therefore, it is hypothesised that aggressive phenotypic changes will be observed under hypoxic conditions (Ruan et al. 2009). In addition, we hypothesise that hypoxia promotes a more mesenchymal phenotype and that reoxygenation significantly affects the EMT status of NSCLC cell lines compared to normoxia. We aim to investigate the correlation between the phenotypic characteristics and gene expression on NSCLC cell lines under varying oxygen concentrations. Our data suggest that reoxygenation promotes a more mesenchymal phenotype in NSCLC cell lines. In addition, over-expression of mesenchymal genes showed the invasiveness of the reoxygenation subpopulation compared to the hypoxic condition. The results from our study highlight the varied expression levels in EMT-associated genes across various oxygen gradients and confirm that dynamics and oxygenation must be taken into consideration in modelling studies.

## Materials and methods

### Cell lines and culture conditions

We have used the following NSCLC cell lines, NCI-H460, HCC827, NCI-H1299, NCI-H1703 and SK-MES-1, which were gifted by Professor Derek Richard (QUT, Brisbane). All of the NSCLC cell lines were STR profiled and authenticated. This gave us the confidence that our cell lines are correctly identified, and not cross contaminated with other cells. Cells were cultured under standard conditions in humidified incubators at 37 °C, 20% O_2_, 5% CO_2_ (normoxia) or in a hypoxic chamber at 1–2% O_2_, 5% CO_2_, (94% N_2_). To avoid the influence of culture media, all culture media conditions were standardised to RPMI-1640-Glutamax (Life Technologies, Inc) supplemented with 10% foetal bovine serum (FBS) (Life Technologies, Inc) and 1% Penicillin–Streptomycin (Life Technologies, Inc).

### Optimisation of culture conditions to investigate hypoxia and reoxygenation effects

**Fig. 1:**
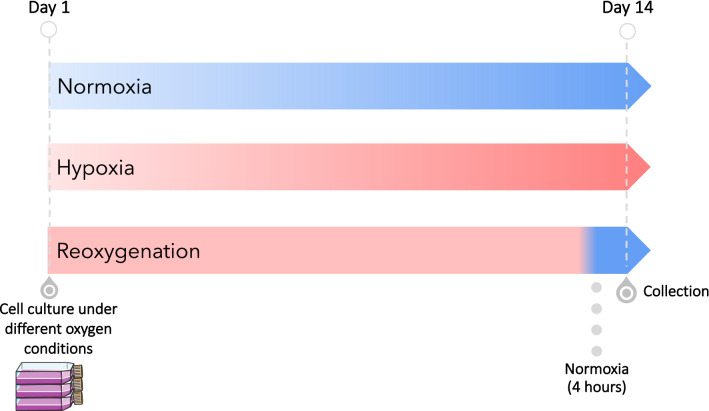
Illustration of the cell culture regimen to study the effects of different oxygen conditions (hypoxia/reoxygenation) in five NSCLC cell lines, NCI-H460, HCC827, NCI-H1299, NCI-H1703 and SK-MES-1

To determine the effects of oxygenation on NSCLC cell lines, cells were incubated at different oxygen concentrations: normoxia (20% oxygen) or hypoxia (1% O_2_) for 14 days to capture *in-vivo* conditions of CTCs as they enter circulation. In addition, cells maintained in hypoxia were further exposed to a pulse of reoxygenation (normoxia) for 4 h prior to collection. This is to model exposure of CTCs to relatively high O_2_ levels while in circulation (Bartkowiak et al. 2020) (Fig. 1). NSCLC cell lines were cultured in replicates of three in T75 flasks (Greiner CELLSTAR™ TC), and cells were collected at full density for downstream experiments.

### STR profiling and mycoplasma testing

To detect cross-contamination in NSCLC cell lines, we used a short tandem repeat (STR) profiling technique on the GenePrint^®^ 10 System (Promega, USA) according to the manufacturer’s instructions at the Genomics Research Centre (GRC; Brisbane, Australia). The NSCLC cell lines were processed using the Applied Biosystems^®^ 3500 Genetic Analyzer. Data were analysed using GeneMapper^®^ v5.0 software (Applied Biosystems). Appropriate positive and negative controls were run and confirmed for each sample. Furthermore, mycoplasma testing was also performed in all NSCLC cell lines using the MycoAlert Mycoplasma detection system (Lonza, Switzerland) at the Genomics Research Centre (GRC; Brisbane, Australia). All cell lines were negative for mycoplasma.

### MTT assay for cell proliferation

To determine cell proliferation, NSCLC cell lines were seeded in 24 replicates at 2500cells/well in 96-well flat-bottomed plates (Grenier Bio-One CELLSTAR^®^). Cells were left to settle overnight in a standard incubator at 37 °C, 20% O_2_, 5% CO_2_ for normoxic conditions and 1–2% O_2_, 5% CO_2_, (94% N_2_) for hypoxic conditions. At 24, 48 and 72 h time points, cells were incubated with 3-(4,5-dimethylthiazol-2-yl)-2,5-diphenyltetrazolium bromide (MTT, 5 mg/ml; Promega, Shanghai, China) at a final concentration of 0.5 mg/ml for 4 h as per manufacturer’s instructions. Absorbance was measured using a CLARIOstar Plus microplate reader (BMG Labtech, Germany) at 490 nm absorbance used as the reference wavelength. The assay was performed in three biological replicates.

### Colony-forming assay

The soft agar colony formation assay was performed according to the previously described protocol (Borowicz et al. 2014). Colonies were stained with crystal violet solution and imaged with a Nikon Eclipse TS2 microscope. Colonies were defined as groups of more than 20 cells when counted manually.

### Sphere formation assay

Post trypsinisation, cells were spun down at 1000*g* for 5 min to obtain a pellet. The cell pellet was resuspended in warmed 1 × concentration of Happy Cell^®^ ASM (Vale Life Sciences) diluted with RPMI-1640-Glutamax supplemented with FBS, and Pen Strep described above. Seeding density was optimised to 0.5 × 10^6^/well for each cell line and plated in 96 well Ultra-Low Attachment plates (Corning). Cells were incubated for 72 h in normoxia and hypoxia (1% O_2_) and visualised with a Nikon Eclipse TS2 microscope.

### RNA extraction and cDNA synthesis

For quantitative mRNA analysis, total RNA (10^6^ cells) was extracted using the RNeasy Mini Kit following the manufacturer’s protocol. RNA extraction procedure comprised of an on-column DNase treatment using RNase-free DNase (Qiagen, Germany), as described by the manufacturer. Total RNA was eluted in 25 μL nuclease-free water. The RNA concentration and quality were assessed using the NanoDrop ND-1000 spectrophotometer (Thermo Fisher Scientific, USA). Purity of samples was estimated by measuring A260/280 and A260/230 ratios of spectrophotometric absorbance to check for copurified contaminants during RNA isolation. cDNA synthesis and genomic DNA elimination were conducted using the RT^2^ First Strand Kit (Qiagen, Germany) according to the manufacturer’s instructions, using 400 ng total RNA for each sample. Negative reverse transcription (without the reverse transcriptase enzyme) was conducted to prove that the samples are devoid of genomic DNA.

### EMT array

Human Epithelial to Mesenchymal Transition (EMT) RT^2^ Profiler PCR Array (Qiagen, Germany) was used to investigate the expression of multiple EMT-related genes in NSCLC cell lines under normoxia and hypoxic conditions. The epithelial to mesenchymal transition RT2 Profiler PCR Array includes cell surface receptor, extracellular matrix and cytoskeletal genes mediating cell adhesion, migration, motility, and morphogenesis. The array also includes genes controlling cell differentiation, development, growth, proliferation and signal transduction and transcription factor associated with the process of EMT. The expression of 84 genes was quantified by qPCR in cells grown in normoxia and hypoxia for 14 days and compared to the same cell types subjected to a pulse of reoxygenation for 4 h. All analyses were performed using the web-based PCR Array Data Analysis Software tool provided on the Gene Globe Data Portal (GeneGlobe 2020). Arithmetic means of the housekeeping genes B2M and RPLP0 were used for normalisation, and fold change was used to analyse differences in gene expression.

### Quantitative real-time PCR (qPCR)

HIF-1α expression levels were tested using primers (fw_GCACAGGCCACATTCACG, rev_TGAAGATTCAACCGGTTTAAGGA) obtained from the literature (Wei et al. 2013). Human β-actin (Forward: CAC CAT TGG CAA TGA GCG GTT C; Reverse: AGG TCT TTG CGG ATG TCC ACG T) was used for normalisation of total RNA input per qPCR reaction. Total RNA (20 ng) was used with PowerUp^™^ SYBR^®^ Green Master Mix (Applied Biosystems, Thermo Fisher Scientific, USA). qPCR conditions were as follows: 50 °C for 2 min; 95 °C for 10 min; 40 cycles at 95 °C for 15 s and 60 °C for 1 min; and a final melting curve analysis with the following conditions: 95 °C for 15 s, 60 °C for 1 min, and 95 °C for 15 s. qPCR reaction per NSCLC cell line under a condition was run in duplicate using the QuantStudio^™^ 7 Flex Real-Time PCR System (Applied Biosystems^™^).

### Western blot analysis

Total protein concentration from cell lysates was quantified using Pierce™ BCA Protein Assay Kit (Thermo Fisher Scientific). Equal amounts of proteins (25 µg) were loaded into 10% SDS-PAGE gels and run at 100 V for 90 min. Proteins were transferred onto a polyvinylidene difluoride (PVDF) membrane at 100 V for 90 min at 4 °C. The membrane was blocked for 1 h at room temperature (RT) using 5% bovine serum albumin (BSA). Membranes were incubated overnight at 4 °C with the following primary antibodies: EGFR (Cell signaling—#4267), HIF-1α (Sigma-Aldrich- # HPA000907), AKT (Cell signaling—#4691P), p-AKT (Cell signaling—#4060), Vimentin—(Santa Cruz—-#sc32322), Pan-Cytokeratin (Thermo Fisher—#ma513203), 4EBP1—(Cell signaling—#9644), p4EBP1—(Cell signaling #2855). Following primary antibody incubation, membranes were incubated with anti-rabbit IgG-HRP secondary antibody (Cell signaling—#7074) or anti-mouse IgG-HRP secondary antibody (Cell signaling—#7076) for 1 h at RT. The membranes were incubated with Pierce ECL Western Blotting substrate (Thermo Fisher Scientific) and imaged using ChemiDoc XRS + System (Bio-Rad Laboratories).

### Statistical analysis

All statistical analyses were performed using GraphPad Prism 8 software version 8.43 (GraphPad Software Inc., La Jolla, CA, USA). For cell proliferation assay and colony-forming assay, an unpaired two-tailed Student’s *t* test was performed. For analysing the EMT array and qPCR, a One-Way Analysis of Variance (ANOVA) with Multiple Comparisons was performed. Data are shown as mean ± standard deviation unless otherwise specified.

## Results

### Cellular proliferation of NSCLC cell lines was not altered under hypoxic conditions

**Fig. 2:**
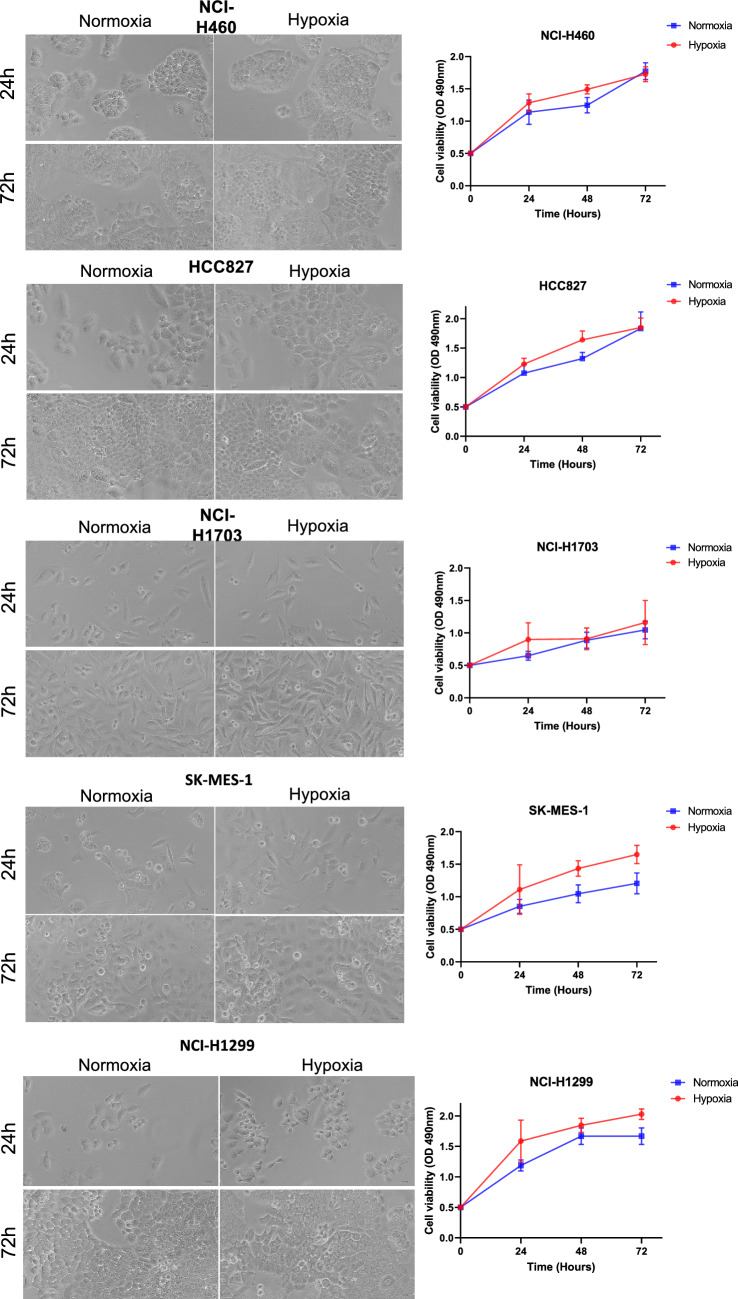
Cell proliferation was assessed by MTT assay using five non-small cell lung cancer cell lines (NCI-H460, HCC827, NCI-H1703, SK-MES-1 and NCI-H1299). Representative images of cells cultured in normoxia and hypoxia for 24 and 72 h are shown on the left panel, and cell viability graphs on the right panel. Data were analysed using paired two-tailed Student’s t-test and shown as mean ± standard deviation

Cell proliferation is a hallmark of cancer and is considered as an important cellular process leading to tumour development (Hanahan and Weinberg 2011). For most cell types, hypoxia induces a decrease in cell proliferation (Hubbi and Semenza 2015). However, certain cell populations maintain cell proliferation in the presence of hypoxia. There were no significant changes observed in cellular proliferation for the five NSCLS cell lines when cultured in hypoxic conditions for 24, 48 and 72 h (Fig. 2) compared to normoxia conditions. Similar findings have been observed in the literature (Ruidong Ma 2018).

### Hypoxia enhances in vitro formation of tumour-spheres and accelerates clonogenicity of non-small cell lung cancer cell lines

**Fig. 3:**
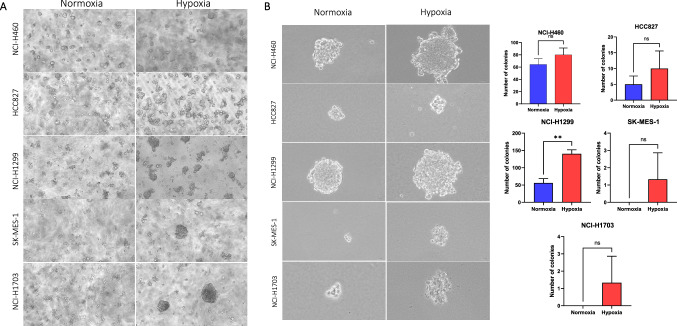
Tumour-initiating ability of non-small cell lung cancer (NSCLC) cell lines. **A** Representative images of tumour-spheres forming ability of NSCLC cell lines under normoxia versus hypoxia-exposed conditions. **B** Representative images and graphs of clonogenic assay of NSCLC cell lines grown in normoxia and hypoxia. Results are shown from three independent replicates for NCI-H460, HCC827, NCI-H1299, SK-MES-1 and NCI-H1703 cells. Data were analysed using paired t-test and shown as mean ± standard deviation

A sphere-forming culture assay was performed to test the tumour-initiating ability of NSCLC cell lines exposed to hypoxia and normoxic conditions. Cells were plated at low density (1x) in Happy Cell^®^ culture medium and cultured under normoxic and hypoxic conditions. Happy Cell^®^ is a liquid matrix that has low viscosity and suspends cells permanently, enabling cells to grow as spheres (Kulasinghe et al. 2015). Representative images were captured following incubation for 72 h to visualise the growth of tumour-spheres (Fig. 3a). On average, hypoxia increased the spheroid formation ability of all investigated NSCLC cell lines (Fig. 3a). In addition, a clonogenic assay was performed by plating cells at a low density in soft agar and observing the ability of single cells to grow into a colony under normoxic and hypoxic conditions. Following 20 days of seeding cells, all colonies (groups of more than 20 cells) were stained and counted. Representative images are shown in Fig. 3b. A higher colony-forming ability trend was observed in cells grown under hypoxia in all NSCLC cell lines investigated, with a statistically significant (*p* < 0.05) difference observed for NCI-H1299 (Fig. 3b).

### Hypoxia promotes EMT in non-small cell lung cancer cell lines

**Fig. 4:**
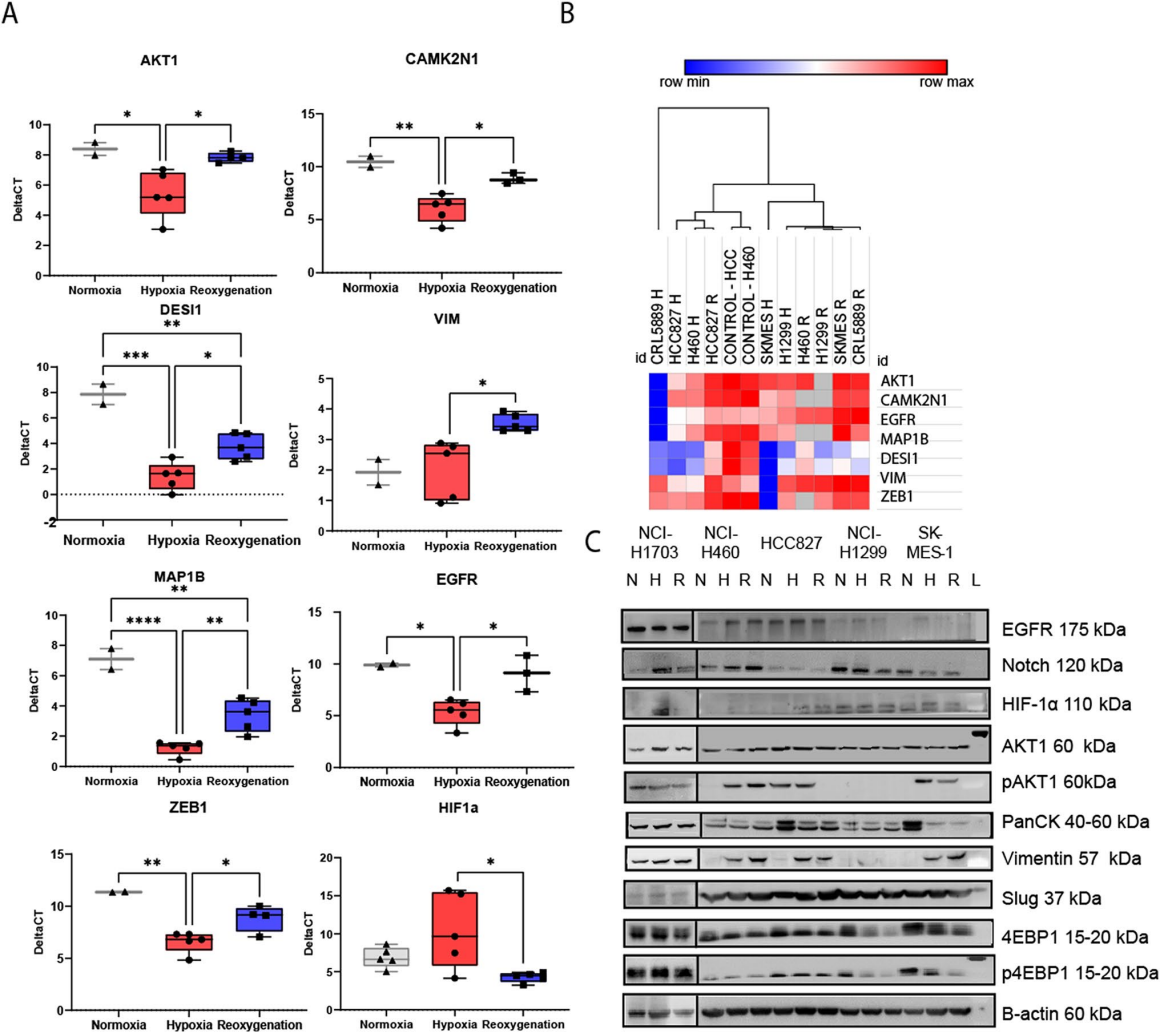
Analysis of EMT-related gene expression levels in non-small lung cancer cell lines (NSCLC) using Epithelial to Mesenchymal Transition RT2 Profiler PCR Array (Qiagen) following exposure to normoxia, hypoxia and pulse of reoxygenation. A) Differential expression of EMT-related genes between normoxic (grey), hypoxic (red) and reoxygenation conditions (blue). B) Hierarchical clustering of epithelial and mesenchymal genes in the NSCLC cell lines. Ct values and fold expression were calculated by the web-based PCR Array Data Analysis Software tool provided on the Gene Globe Data Portal (GeneGlobe 2020), and the clustering was generated using Morpheus (available at https://software.broadinstitute.org/morpheus). C) Western blot results of EGFR, Notch, HIF-1α, AKT1, pAKT1, Pan-CK, Vimentin, Slug, 4EBP and p4EBP protein levels across all five NSCLC cell lines under normoxia, hypoxia and reoxygenation. *N* normoxia, *H*  hypoxia, *R* reoxygenation, *L* molecular weight ladder

To investigate the influence of hypoxia on the expression of genes associated with EMT, we have used a commercially available EMT array, and the mRNA expression changes were verified at the protein level using western blotting. We compared the mRNA expression changes of 84 EMT genes in five NSCLC cell lines under normoxia, hypoxia and reoxygenation. We found that the mRNA expression levels of seven EMT-related genes (AKT1, CAMK2N1, EGFR, MAP1B, DESI1, VIM and ZEB1) were significantly altered across the different oxygen conditions (Fig. 4a). Furthermore, we have analysed HIF1α expression levels using qPCR (Fig. 4a). HIF-1α is a factor that regulates a number of genes that promote the adaptation to hypoxia. As expected, although not significant, we observed an increase in the expression of HIF-1α under hypoxic conditions compared to normoxia and a significant (*p* < 0.05) decrease in the expression of HIF-1α after reoxygenation in comparison to hypoxia.

Other studies have shown that EMT results in transcriptional inhibition and/or loss of epithelial markers such as E-cadherin and cytokeratins and gain of mesenchymal markers such as vimentin, N-cadherin and fibronectin (Craene and Berx 2013; Aiello et al. 2018). The mRNA expression levels of vimentin were increased in NSCLC cell lines under hypoxic conditions when compared to normoxic conditions (Fig. 4a). The expression of vimentin was significantly upregulated after exposure to reoxygenation (Fig. 4a). In lung cancer, increased expression of vimentin is used to identify the progression of epithelial cells from a localised lesion to invasive, metastatic tumour cells (49–51). In addition, protein levels of nine targets (EGFR, Notch, HIF-1α, AKT1, pAKT1, Pan-CK, Vimentin, Slug, 4EBP and p4EBP) were investigated using western blotting. In specific cell lines (NCI-H460, HCC827 and SK-MES-1), we observed an increase in vimentin protein levels and a decrease in pan-cytokeratin (Fig. 4). The switch of pan-cytokeratin/vimentin expression implies that EMT might have occurred under hypoxic conditions.

### Cellular responses to hypoxia and reoxygenation in non-small cell lung cancer cell lines

After exposure to a pulse of reoxygenation, ZEB1 expression was significantly upregulated in NSCLC cell lines under normoxia and reoxygenation compared to hypoxia. In our study, AKT1 mRNA levels were significantly downregulated in NSCLC cell lines in hypoxic conditions compared to normoxic conditions, and similar AKT1 mRNA expression levels were found between normoxic and reoxygenation conditions (Fig. 4a). In contrast, AKT1 at the protein level was not altered between conditions. pAKT1 showed relatively high levels of protein abundance after hypoxia and reoxygenation compared to normoxia, but only for NCI-H460 and SK-MES-1 cell lines (Fig. 4c). We found no significant differences at the protein level for EGFR after reoxygenation compared to normoxia for NSCLC cells.

## Discussion

The control of O_2_/CO_2_ concentrations and temperature are essential to maintain cell homeostasis, and minor changes to this can affect cell phenotype and gene expression (Cummins et al. 2020). Hypoxia activates complex transcriptomic changes of several downstream signalling pathways, with very little knowledge as to how cells (e.g. CTCs) activate these pathways in response to hypoxia (Harris 2002; Ameri et al. 2010; Donato et al. 2020). The use of hypoxic culture conditions is, therefore, significant for studying the effects of hypoxia on CTCs.

In this study, we have established an in vitro model system that recapitulates CTCs intravasation in NSCLC patients to mimic in vivo conditions. We observed no alterations in the cellular proliferation of NSCLC cell lines in hypoxic conditions. In contrast, a previous study has shown a significant increase in the proliferation of A549 cells under hypoxic conditions compared to normoxia (Hu et al. 2018). This difference may arise due to the differences in the cell lines investigated (A549). Hypoxia has been shown to inhibit cell proliferation in many cell types, and overexpression of HIF-1α alone is insufficient to induce cell cycle arrest (Hubbi and Semenza 2015). In contrast, many cancer cells, even in the presence of elevated HIF-1α levels, have been shown to maintain proliferation (Zhong et al. 1999). Similarly, we have observed in our spheroid formation assay that hypoxia increased the proliferation of NSCLC cell lines.

There are currently limited studies investigating the influence of reoxygenation on tumour development. To explore this phenomenon, we modelled the reoxygenation conditions that tumour cells experience in the bloodstream. We cultured NSCLC cell lines under hypoxia for 14 days and then exposed cells to a pulse of reoxygenation for 4 h. After reoxygenation, eight EMT-related genes (AKT1, CAMK2N1, EGFR, MAP1B, DESI1, VIM and ZEB1 and HIF-1α) were differentially expressed across varying oxygen conditions. Except for HIF1α, the rest of the genes were significantly upregulated in reoxygenation compared to hypoxia. Over-expressed mesenchymal genes (CAMK2N1, and VIM) in NSCLC show the invasiveness in the reoxygenation cell subpopulation compared to the hypoxic condition. It has been shown that cells in reoxygenation showed overexpression of cell–cell and cell-ECM adhesion genes compared to hypoxic (Zhang et al. 2014). A study has also shown that repeated cycles of hypoxia-reoxygenation may directly regulate the cell cycle, glucose metabolism and angiogenesis (Cui et al. 2012). The varied expression in EMT-associated genes between the three conditions confirms the dynamics of oxygenation, an important aspect that must be taken into consideration in modelling studies.

Complex signalling pathways (Wnt/ β-catenin, TGF-β, EGFR, Notch, Hedgehog) and transcriptional factors (Snail, Slug, Twist and Zeb1/2) initiate and orchestrate the process of EMT. We saw a lower expression of ZEB1 mRNA in NSCLC cell lines under hypoxia compared to normoxia and reoxygenation. In contrast, exposing cells to hypoxia has been shown to increase EMT-promoting transcription factors such as ZEB1 (Foster et al. 2014) and have been implicated in driving EMT in lung cancer (Larsen et al. 2016). We have seen that vimentin at both mRNA and protein levels was upregulated in NSCLC cell lines in hypoxia compared to normoxia. Similarly, AKT1 protein levels were higher in hypoxic conditions compared to normoxia and reoxygenation. AKT1 has been identified as a candidate driver gene in adenocarcinoma (Foster et al. 2014) and has been identified as an important regulator of invasion and metastasis of lung cancer cells having KRAS or EGFR mutations (Rao et al. 2017). AKT1 kinase binds to and phosphorylates vimentin, preventing vimentin from caspase-induced proteolysis and ultimately leading to an increase in motility and invasiveness, as well as enhanced tumour growth and metastasis (Kidd et al. 2014). Under hypoxic conditions, EGFR protein levels were relatively higher in NSCLC cell lines compared with the other two conditions. Overexpression of EGFR is implicated in the pathogenesis of many human cancers, including NSCLC (Bethune et al. 2010) and has been known to correlate with poor prognosis (Ohsaki et al. 2000). Treatment with EGFR tyrosine kinase inhibitors (EGFR-TKIs) has been the first-line treatment for NSCLC patients who harbour activating EGFR mutations, however, often acquired resistance to EGFR TKIs, which is common within 12 months of treatment, mostly due to the T790M mutation detected in approximately 60% of patients (Rosell et al. 2012; Cortot and Janne 2014; Sacher et al. 2014). The acquisition of mesenchymal morphology in relapsed tumours is distinct from EGFR-TKI resistance (Tulchinsky et al. 2019).

The findings of this pilot study have some limitations. It is nearly impossible to establish the time that CTCs in circulation have been subjected to hypoxic conditions prior to release into the bloodstream from primary or metastatic sites. Furthermore, tumour microenvironment conditions are difficult to mimic and make the current study an approximation in identifying oxygenation effects on proliferation, colony and sphere formation, as well as the impact on EMT. Other cultural conditions should also be accounted for, such as pH changes. Additional evaluation of other phenotypic assays are needed, including migration and invasion assays and further exploring proliferation rates and cell cycle. Future studies with patient samples could utilise this model to understand better the effects of oxygenation on tumour cells and their ability to survive and extravasate.

Our data suggest that when investigating CTCs as a prognostic biomarker in NSCLC, it is also important to take into consideration mesenchymal markers to obtain a comprehensive overview of the status of CTC in circulation. We also suggest that reoxygenation increases the expression of a number of EMT markers, leading to a more mesenchymal phenotype.
